# Analyzing the land and labour productivity of farms producing renewable energy: the Italian case study

**DOI:** 10.1007/s11123-023-00659-2

**Published:** 2023-01-21

**Authors:** Antonella Basso, Maria Bruna Zolin

**Affiliations:** Department of Economics, Ca’ Foscari University of Venice, Cannaregio 873, Fondamenta San Giobbe, 30121 Venice, VE Italy

**Keywords:** Q01, Q42, Q56, Land and labour productivity, Fixed factor remuneration, Renewable energy production, Agriculture, Solar and biogas energy, Regression analysis

## Abstract

The paper computes and analyses some relevant indicators of economic performance of Italian farms producing/not producing renewable energy, and compares the economic results of the two set of farms. The source of data is the European Farm Accountant Data Network; the farms belonging to this network are analysed in relation to their structural differences, type of farming, geographical areas, economic size, as well as the type of renewable energy produced. After an in-depth statistical investigation, the main economic ratios are computed and analysed using also multivariate regression models, with a special focus on the production of solar and biogas energy. In terms of land and labour productivity and fixed factor remuneration, the results show that farms producing renewable energy perform better than the other farms. This positive effect is particularly accentuated in large companies that produce biogas, followed by farms that produce solar energy. There are still many obstacles that limit the production of renewable energy in agriculture; among these, still insufficient research and information on best practices in agriculture and, in Italy, the complexity and dispersion of the institutional legislative framework and of the public support systems. However, the need to increase the production of renewable energy has become a priority for many European countries both in the short- and in the medium term, especially in light of recent events related to the war in Ukraine.

## Introduction

The main aim of this contribution is to analyse some relevant indicators of economic performance of farms that produce renewable energy (RE) and to compare the economic results obtained by RE producers with those of farms that do not produce it in Italy.

According to the World Energy Outlook 2019 (IEA (2019)), oil markets and geopolitical tensions, carbon emissions and climate targets, lack of access electricity for a still important part of the global population (estimated at 850 million people) and the progressive growth of the world population, characterise the world energy sector. Despite the growth in low-emission energy production, the Middle East still represents the region that supplies the largest share of oil on whose imports some important economies depend, especially those of the EU and some Asian countries. Besides, many EU countries largely depend on Russia for the supply of natural gas, and recent events related to the war in Ukraine have dramatically highlighted the need to reduce this dependence in a short time.

In addition, the growth in energy demand from developed and emerging countries have led to a progressive depletion of easily accessible fossil energy sources and, combined with the progressive increase in waste produced in rich countries, to an increase in greenhouse gas emissions (especially CO_2_). For this reason, the Kyoto Protocol has imposed limits on industrialised countries in terms of greenhouse gas emissions, and many countries have adopted measures aimed at reducing the energy dependence on fossil sources. Indeed, ensuring the access to affordable, reliable, sustainable and modern energy for all is goal 7 of the UN (2018) sustainable development goals.

Energy produced from renewable sources is undoubtedly a solution contributing to reduce these concerns. According to the International Energy Agency (IEA), in 2018, 13.5% of the energy produced globally came from renewable energy sources, which include hydroelectricity, biofuels, renewable municipal waste, solar photovoltaic and thermal, wind, geothermal and tide. From 1990 to 2018, renewable energy sources have grown on average by 2.0% per year (IEA (2020a)).

In 2018, in the EU countries, the share of energy consumption from renewable sources was 18% of the total, 17.8% in Italy (GSE (2020)). For 2030, the Italian Integrated National Plan for Energy and Climate (MISE et al. (2019)), sets out a share of renewable energy on total consumption of 30%.

According to the Agenzia Nazionale Efficienza Energetica (ENEA), the Italian agri-food system is energy-intensive and in 2016 it ranks fourth after mechanics, steel and chemistry in the ranking of sectors with the highest energy consumption (ENEA (2018), page 41). It absorbs 11.3% of the total consumption of electricity and liquefied petroleum gas (LPG) and 7.8% of diesel, with a preponderant weight of agriculture compared to agro-industry. Consequently, it is an area where significant improvements are possible, both in the consumption patterns and in the contribution to the production of energy from renewable sources.

The research question of this paper is to investigate if farms producing renewable energy as related agricultural activity obtain a higher economic outcome/performance with respect to farms not producing RE. This issue is relevant, since a higher economic outcome could convince more farmers to introduce the production of renewable energy, thus contributing to the energy security goal and to the reduction of CO_2_ emissions, as well as to the reduction of the Italian dependence on imports. Moreover, we compare the economic performance obtained by farms producing renewable energy from different sources, identifying the energy production sources that better reward the fundamental productive factors, i.e. land and labour. To our knowledge, such a comparison is less explored in the literature and represents a relevant contribution. Note that the results of the analysis can be useful in order to boost the energy production by the agricultural sector, so that this sector can effectively help to meet the EU energy targets.

To answer the question, we analyse the Italian farms belonging to the FADN network in 2018, taken as a representative year, and compare the companies not producing energy from renewable sources with those that produce it. The differences in structural terms, in terms of the type of farming, geographic areas, economic dimension, as well as of the type of renewable energy produced, are studied. The productivity and the remuneration to the fixed factors of land and labour are then examined and discussed in detail. The results represent a tool that can be useful at the macro level for policy makers, and at the micro level for companies, mainly in energy importing countries, such as Italy and several other European countries. For these countries the recent explosive increases in the price of fossil fuels, specifically oil and natural gas, are fueling inflation, thus eroding the spending power of citizens, already reduced by the consequences of the COVID-19 pandemic, and slowing the ongoing economic growth. On the other hand, the strong increase in the price of energy will likely advantage the producers of renewable energies (IEA (2020b); for additional remarks see IEA (2021), Wang et al. (2014) and Fernandez-Perez et al. (2016)).

The paper is organised as follow. Section *Background* briefly presents the background of renewable energies in agriculture, in the EU and in Italy. In Section *Data and methodology* we present the methodologies used: statistical comparisons, the computation of economic ratios and multivariate regression models. Section *Statistical analysis* reports the data used, and Section *Economic analysis* describes the sample and the statistical analysis carried out. Section *Multivariate regression analysis* reports and discusses the results of the economic analysis performed, focused on an analysis of productivity and remuneration to the fixed factors. Section *Conclusions* discusses the results of the multivariate regression analysis carried out. Finally, Section *Conclusions* presents the main conclusions of the study and some policy implications.

## Background

As is known, in the Paris agreement the European Commission has declared that the EU aims to be climate neutral, with zero green house gas emission, by 2050. In order to reach this ambitious goal, a set of measures have to be implemented and/or strengthened. Indeed, the EU for years has been putting a lot of attention to “green” issues and to renewable energies. At the end of 2018, the EU directive 2018/2001 on renewable energies entered into force as part of the “Clean energy for all Europeans" package, in application of the commitment made in the Paris agreement to reduce CO_2_ emissions. It replaces the previous directives in energy from renewable sources (Directive 2009/28/ EC, repealing Directives 2001/77/ EC and 2003/30/ EC). The directive 2018/2001 sets for 2030 the goal that at least 32% of final energy consumption comes from renewable sources, with a clause for a possible upward revision by 2023, and a 14% increase in the share of renewable fuels in transport (European Parliament (2020)).

In January 2020, the European Parliament (Zygierewicz and Salvador Sanz (2021)) adopted the resolution on the European Green Deal proposed by the European Commission in 2019 in which the role of renewable energies in the transition to a zero-emission economy is among the main objectives. The Parliament resolution calls for the 2018 directive to be amended with the introduction of stricter and more ambitious targets. The European Commission on 14th July 2021 should present a proposal to revise the Renewable Energy Directive to align the European objectives with the Green Deal and be able to reduce CO2 emissions by 55% by 2030. According to the draft, the share of renewables in the EU energy mix is expected to rise from the current 20% to 38–40% by 2030 (Taylor and Iorio (2021)).

According to the European Commission (2018), in 2016 the contribution of the agricultural sector to the EU’s total greenhouse gas emissions is almost 10%. By 2030, EU emissions in agriculture should be reduced by 30%, and the share of renewable energies should be at least 32%. The production of renewable energy on farms can strongly contribute to the reduction of these emissions (Liu et al. (2017), Martinho (2018), Eyuboglu and Uzar, U. (2020), Rokicki et al. (2021)). Moreover, REs in agriculture can improve energy security and boost the development in rural areas (see Rikkonen et al. (2019)), besides providing an integration of agricultural incomes.

The Common Agricultural Policy (CAP) contributes to climate mitigation with specific measures within two pillars. In the first pillar, it does so through the mandatory instrument of cross compliance, which includes environmental requirements and obligations to be respected in order to receive direct aid, and green payments covering a wide geographical range of agricultural products. On the other hand, rural development (the second pillar) operates mainly through specific voluntary measures and plays an important role in achieving the environmental objectives of the CAP. The debate on the “new” CAP is proceeding slowly for several reasons, including the coronavirus emergency and the long debate on the Multiannual Financial Framework 2021–2027. To this end, the Commission has proposed a 2-year transitional regime, which started on 1st January 2021. The European Commission’s legislative proposals for the post-2020 CAP require green payments to be maintained but included as part of the new cross compliance requirements. A new environmental instrument would be introduced in pillar 1, the “eco-scheme”, and the CAP would be implemented through national strategic plans offering Member States ample flexibility (European Commission (2019)). The “new” strategic plans will include the ambitions of the European Green Deal, in particular the Farm to Fork strategy (European Commission (2020)). Overall, 40% of the total CAP expenditure will be dedicated to climate actions including the production and consumption of REs; the two pillars may include also aid to renewable energies, mainly through measures provided for by the national regulations.

In the Farm to Fork strategy, circular economy is an essential issue (IRENA and FAO (2021)); on this regard, biogas is a primary driver of the circular economy at the local level. It helps farmers to drastically reduce the negative externalities of their activities by allowing them to recycle organic nutrients and restore soil fertility, providing in addition RE.

Let us now turn our attention to Italy. According to ISPRA (Higher Institute for Environmental Protection and Research) data (ISPRA (2020)), in Italy greenhouse gas emissions attributable to the agricultural sector derive about 80% from livestock farms, 15% from the use of synthetic nitrogen fertilisers and other nitrogen inputs to the soil, and another 5% from rice cultivation. Furthermore, the analysis of ISPRA data shows that overall emissions in agriculture decreased by 13% compared to 1990 levels. This is due to the reduction both in the number of reared animals and in the agricultural areas and production, to the reduction of fertilisers used, thanks to changes introduced in the management of agricultural techniques, and to the implementation of the interventions of the CAP.

In Italy, according to GSE (Gestore dei Servizi Elettrici, namely Energy Services Manager) data (GSE (2019)) it emerges that in the agricultural and forestry sector, in 2018, the production of renewable energy contributes for 50% of energy consumption of RE and for 8.7% of the total energy consumption. A fundamental role is played by biomasses which, despite being reproducible and inexhaustible resources, must follow the principles of sustainability, in order not to alter ecosystems and avoid conflicts in land use (Ballarin et al. (2011), Chel and Kaushik (2011), Popp et al. (2014), Waheed et al. (2018), as well as in the production of solar or wind energy. According to Rocca (2021), in Italy in 2021 photovoltaics produce about 21,000 MW, using around 40,000 hectares, of which less than half are on agricultural land and the rest are placed on the roofs of houses, sheds and shelters. To double the installed capacity by 2030, estimates call for an additional 40,000 hectares to be used. On the other hand, this is <1% of the difference between the total agricultural area (17.4 million hectares) and the Utilised Agricultural Area or UAA (12.9 million hectares) of Italy. In these terms, a serious conflict between food security and energy security does not seem to exist. It should be emphasised, however, that there are cases in which photovoltaic systems are located in fertile and valuable agricultural areas. The problem is how to drive the choices of the sites on which to locate the interventions in a sustainable way. With respect to this, in Italy the law (specifically the DM 10.09.2010, Ministero dello Sviluppo Economico (2010)) requires the Regions to indicate areas and sites that are not suitable for the installation of plants for the production of renewable energy. Only a few regions, however, have applied this legislation to date. Of course, the issue of the competition on the usage of land between food and energy security is of the utmost importance and cannot be negletted.

In Italy, public support to the installation of plants and the production of renewable energy is characterised by different and complex mechanisms, which consider several factors such as the type of source, the size of the plant or the date of construction. They can be summarised in 3 broad categories: green certificates, feed-in tariff, and grants and loans for the construction of the plant (Carrosio (2014)).

According to Bartolini et al. (2017), the current incentive mechanism does not allow the achievement of EU energy objectives at local level and the incentive tools need to be tailor made according to the socio-economic and environmental regional conditions. Moreover, their results show that agri-food energy production can help farmers stabilise their income and maintain viable rural areas. In their paper on biogas production and consumption in Italy, Pirelli et al. (2021) underline the needs for policies designed on a local scale to reduce the potential risks of environmental impact linked to biogas, and to strengthen successful management practices.

As far as the agricultural sector is concerned, these productions also benefit from a fiscal treatment on the side of direct income taxes. On the other hand, there are numerous restrictions in agricultural areas for obtaining incentives, especially in the case of the installation of photovoltaic systems which, if located in agricultural areas, cannot benefit from public incentives. The plants to produce solar energy are classified into 3 categories, based on the potential of the plant. The subsidy is decreasing, the highest being paid to small plants. In Europe, Italy ranks second in the list of countries for installed solar photovoltaic (PV) capacity (Di Nucci and Prontera (2021)). However, the achievement of some of the European 2020 targets is due more to the effects of the economic crisis than to the application of policies to support renewable energy and energy efficiency.

In the case of biogas, the incentive is increased by a variable bonus linked to the removal or not of nitrogen, solid or liquid agricultural waste used as fertiliser. The transformation of livestock manure into renewable energy is a good example of a circular economy. This is particularly important in areas vulnerable to nitrates of agricultural origin (Musacchio et al. (2020)), which must respect the ceiling of 170 kg/hectare/year (average quantity of nitrogen introduced by the company; however, some Italian regions have obtained a derogation that allows the use of up to 250 kg/hectare/year of nitrogen). Even in areas not vulnerable to nitrates, the amount of total nitrogen in the field brought by animal manure must not exceed 340 kg. per hectare and per year. Finally, the treatment of animal waste is necessary to avoid polluting soil and water.

## Data and methodology

The main aim of this contribution is to analyse the production of renewable energy in agriculture and investigate whether and when it is profitable for farmers.

In order to answer this question, we apply three different methods. But first let us introduce the data that will be used in the empirical analysis.

### Data

The source used for the data is the FADN (RICA^1^ for Italy), established in 1965 with Regulation (EEC) no. 79/65/EEC in order to assess the structure and performance of EU farms and to measure the impacts of the CAP. The data collection is conducted by each Member State and, to ensure representability, random sampling is adopted.

For Italy, a stratified random sampling is used since 2003, based on three variables: geographical location, economic dimension and technical economic orientation, derived from the previous census data. The participation of Italian farmers is mandatory and a series of non-monetary incentives are envisaged for participants. On average, the Italian FADN sample includes about 11,000 companies and ensures excellent coverage (95% of the Utilised Agricultural Area or UAA, 97% of the value of Standard Output or SO, 92% of work units, 91% of the livestock units).

The collected data is high quality microdata, but the database is not exempt from some limitations. The Italian farms surveyed are farms with a production, measured in European Size Unit (ESU), exceeding 8000 euros and with UAA >1 hectare. However, these types of farms represent an important reality in the Italian context for their number (about 50% of the total) and presence on the territory and the sample describes a non-subsistence agriculture. The observation year taken into consideration in the analysis is 2018, taken as a representative year.

The analysis compares farms producing energy from renewable sources with those not producing renewable energy, with respect to several variables:

- Type of farming (crop, livestock, mixed)
- Management system
- Legal status
- Educational level
- Age (under/over 40 years old)
- Utilised Agricultural Area (UAA)
- Annual Work Units (AWU, expressed in full time equivalent, 1800 h per year)
- Standard Output (SO), computed as the standard output resulting from the standard farm activity and the (eventual) production of renewable energy
- Total Revenues (TR)
- Farm Net Value Added (FNVA)
- EU support Pillar 1
- EU support Pillar 2.

We also distinguish these farms by geographic area (Centre, Islands, North-east, North-West, South) and altimetric area (hill, mountain, plain).

In addition, we computed the following economic ratios, to compare the economic outcomes: SO/UAA, SO/AWU, FNVA/UAA, FNVA/AWU.

The FADN data report the production of renewable energy related to biogas, solar, wind, wood energy and to *other sources*. Given the size of the sample and the objective of the research, the analysis will be carried out more in-depth for farms producing renewable energy from solar and biogas. On the other hand, despite their large number, the *other sources* will not be considered in detail because of the lack of specific information on this category, probably due to their heterogeneity. Analogously, as regards the type of farming, the analysis will be carried out more in-depth for crop and livestock farms.

### Methodology

First, in Section *Statistical analysis* we carry out a comprehensive statistical analysis which allows us to compare farms not producing and producing RE from different points of view, as well as to distinguish between different sources of RE.

Secondly, in Section *Economic analysis* we perform an economic analysis of relative indicators related not only to the output obtained by a farm but also to the production factors that in agriculture cannot be ignore: land and labour.

In the literature different indicators have been proposed to analyse the economic performance (productivity, remuneration to the fixed factors/profitability) in the agricultural sector (Dorward (2013), Ball et al. (2015)), according to the aims of the analysis, the territorial level, the data source and the methodology applied.

We may cite, among others, the following analysis carried out at a territorial level: Sharma et al. (1990), that compares developed and developing countries, Gorton et al. (2005), that compares “old” and “new” EU member states, Blazejczy-Majka et al. (2012), that also considers “old” and “new” EU regions, Marongiu and Cesaro (2017), that focuses on Italian inner areas, Giannakis and Bruggeman (2018), that investigates the labour productivity in the six main agricultural systems across European NUTS2 regions.

At a micro level, we may cite, among others, the following papers: Coppola et al. (2013) and Coppola et al. (2020), that estimate a profitability index which relates real net income to a reference revenue on Italian farms, Leonardo et al. (2015), that computes total crop production and revenue per hectare for different agricultural systems in Mozambico.

Many of the studies on this subject share the focus on land and/or labour, as main productive factors in agriculture. Indeed, the reference to land and labour is essential and it is crucial to take them into account in agricultural production choices. In this context, the proxies chosen are particularly suited to the purpose of our analysis and shed light on performance indicators that may be of help to show that the production of RE may be convenient and thus boost the supply of energy from renewable sources in agriculture.

The economic indicators chosen for this analysis are listed in Table 1. More specifically, we compute the standard output per hectare and per annual work unit, SO/UAA and SO/AWU, to analyse the land and labour productivity, and the farm net value added per hectare and per annual work unit, FNVA/UAA and FNVA/AWU, for the remuneration to the fixed productive factors.

**Table 1:** Performance indicators

Performance indicator	Detailed description	Used for:
SO/UAA	Standard output (euros) per 1 hectar of Utilised Agricultural Area	Land productivity
SO/AWU	Standard output (euros) per 1 work unit	Labour productivity
FNVA/UAA	Farm net value added (euros) per 1 hectar of Utilised Agricultural Area	Fixed factor land
FNVA/AWU	Farm net value added (euros) per 1 work unit	Fixed factor labour

In the literature different indicators have also been proposed; for example, the value added and/or the farm net income could be used in place of the farm net value added. We chose these ratios because they measure the ability of the productive process to remunerate all the fixed productive factors and the entrepreneurial activity, and they are a good approximation of the production and income per hectare and per worker.

Thirdly, in Section *Multivariate regression analysis* we consider two multivariate regression models to take into account the relationship between SO or FNVA, the output indicators analysed, and the main productive factors simultaneously.

The multiple regression models allow also to take into account different characteristics of farms through the use of specific dummies, listed in Table 2. The characteristics accounted for are: the type of farming, the altimetric area, the source of renewable energy produced, the management system, the educational level, the age, the EU support (Pillar 1 and Pillar 2) and the geographical area.

**Table 2:** Dummy variables included in the regression models

Dummy	Variable	value = 0	value = 1
*d*_1_	RE production: biogas	Not producing biogas	Producing biogas
*d*_2_	RE production: solar	Not producing solar	Producing solar
*d*_3_	RE production: wind	Not producing wind	Producing wind
*d*_4_	RE production: wood	Not producing wood	Producing wood
*d*_5_	RE production: other RE	Not prod. other RE	Producing other RE
*d*_6_	Altimetric area: hill	Otherwise	On the hill
*d*_7_	Altimetric area: mountain	Otherwise	On the mountain
*d*_8_	Type of farming: livestock	Crop or mixed	Livestock
*d*_9_	Type of farming: mixed	Crop or livestock	Mixed
*d*_10_	Management system	Direct	Other man. syst.
*d*_11_	Educational level: upper secondary	Otherwise	Upper secondary
*d*_12_	Educational level: tertiary	Otherwise	Tertiary
*d*_13_	Age	Over 40 years old	Under 40 years old
*d*_14_	EU support: (0,10] thousand €	Otherwise	in (0,10]
*d*_15_	EU support: (10,25] thousand €	Otherwise	in (10,25]
*d*_16_	EU support: > 25 thousand €	Otherwise	>25
*d*_17_	Geographical area: Centre	Otherwise	in Centre
*d*_18_	Geographical area: Islands	Otherwise	in Islands
*d*_19_	Geographical area: North-East	Otherwise	in North-East
*d*_20_	Geographical area: South	Otherwise	in South

The model for the standard output can be written as:

1 $${{{{\rm{SO}}}}}_{i}={\beta }_{0}+{\beta }_{UAA}{{{{\rm{UAA}}}}}_{i}+{\beta }_{AWU}{{{{\rm{AWU}}}}}_{i}+\mathop{\sum }\limits_{k=1}^{K}{\beta }_{{d}_{k}}{d}_{k}+{\varepsilon }_{i}\,\,i=1,\ldots ,n$$

where SO_*i*_ is the standard output of farm *i*, with *i* = 1, …, *n*, the *β*s indicate the coefficients associated to the explanatory variables and *ε*_*i*_ is the error term of the regression for farm *i*.

We observe that a variable characterising farms on the plain, for example, is not included in the regression model (1), in order to avoid multicollinearity among the explanatory variables, and of course the same procedure is applied to all the other features taken into account when defining the associated dummy variables, too.

An analogous model is introduced for the farm net value added:

2 $${{{{\rm{FNVA}}}}}_{i}={\gamma }_{0}+{\gamma }_{UAA}{{{{\rm{UAA}}}}}_{i}+{\gamma }_{AWU}{{{{\rm{AWU}}}}}_{i}+\mathop{\sum }\limits_{k=1}^{K}{\gamma }_{{d}_{k}}{d}_{k}+{\varepsilon }_{i}\,\,i=1,\ldots ,n$$

where SO_*i*_ is the standard output of farm *i*, with *i* = 1, …, *n*, the *γ*s indicate the coefficients associated to the explanatory variables and *ε*_*i*_ is the error term of the regression for farm *i*.

The multivariate models (1)–(2) express the combined impact on SO or FNVA, respectively, of the productive factors UAA and AWU, thus overcoming the partial view provided by the use of indicators/ratios. In addition, they also enable us to assess the differences due to the renewable energy produced and to all the other features taken into account.

## Statistical analysis

As mentioned, the selected sample belongs to the FADN network, which in 2018, the year covered by our study, surveyed 10,386 companies located in Italy. After removing the companies with an economic size of <8000 euros and more than 10 million euros, we are left with 9927 companies. Of these farms, 711 companies (7.2% of our sample) claim to have produced energy from a renewable source, namely from biogas, wind, wood-burning and solar or other unspecified sources. The remaining 9216 farms (92.8%) do not currently produce renewable energy, highlighting the huge potential of the agricultural sector to contribute to sustainable growth.

### Type of farming

Table 3 shows the distribution of farms by type of farming. As for the farms which do not produce RE, 2508 are dedicated to livestock farming (27.2%), 6314 to crops (68.5%) and the remaining 394 (4.2%) are classified as mixed. The companies producing RE are distributed as follows: 248 (34.9%) are devoted to livestock, 417 (58.6%) to crops and 46 (6.5%) are mixed. By comparing the relative distributions, we note that farms producing RE are less present in crops and more in livestock with respect to farms not producing RE.

**Table 3:** Distribution of companies by type of farming

Farms	Crop	Livestock	Mixed	Total
*Number*				
Farms not producing RE	6314	2508	394	9216
Farms producing RE	417	248	46	711
Total	6731	2756	440	9927
*Relative distribution*				
Farms not producing RE	68.5%	27.2%	4.3%	100.0%
Farms producing RE	58.6%	34.9%	6.5%	100.0%

Family work prevails in both groups (farms producing/not producing RE), but companies that produce renewable energy show a higher incidence of the direct form, with a prevalence of non-family work. As for legal status, individual companies prevail. The farmers’ educational level is higher, on average, in companies producing RE.

### Renewable energy source

Table 4 reports the number and the relative distribution of farms producing RE from the different sources considered. Solar power production is widespread and involves 372 companies. 40.9% of it (152 farms) are livestock holdings, 52.4% (195 farms) concerns crops and the remainder are mixed. As was only logical to expect, the production of biogas regards mainly the livestock sector.

**Table 4:** Farms producing renewable energy by source

Farms	Crop	Livestock	Mixed	Total
*Number*				
Biogas	10	28	1	39
Solar	195	152	25	372
Wind	12	3	1	16
Wood-burning	6	12	2	20
Other sources	217	68	20	305
*Relative distribution*				
Biogas	25.6%	71.8%	2.6%	100.0%
Solar	52.4%	40.9%	6.7%	100.0%
Wind	75.0%	18.8%	6.3%	100.0%
Wood-burning	30.0%	60.0%	10.0%	100.0%
Other sources	71.1%	22.3%	6.6%	100.0%

Companies that produce energy from wind energy are poorly present (only 16 farms), with crops as the prevailing agricultural system, while in wood-burning (16 farms) livestock holds the primacy (60%). Energy production from other unspecified sources regards mainly crops (217 holdings, 71.1%), followed by livestock (68 holdings, 22.3%) and lastly mixed (20 holdings, 6.6%).

Let us observe that the production of energy from the different renewable sources is not mutually exclusive, and indeed we find a few farms that produce renewable energy from more than one source simultaneously. Moreover, as many as 305 farms producing RE in the FADN data base are included under the label *other sources*; of course, it would be interesting to deepen the investigation on these other sources, if only the disaggregated data were available.

### Geographical area and altimetric area

Sampled companies producing RE are spread across the 5 Italian geographical areas considered, with different intensities. 32.3% of them are concentrated in north-eastern Italy, followed by those located in central Italy (28%) and north-western Italy (25.5%). At a distance are the companies of southern Italy (10.1%) and islands (just 4.1%).

The distribution of farms producing solar energy follows a similar trend: the north-east regions detect the highest frequency (35.2%), followed by those in north-western Italy (29.6%) and in the centre (25.5%). The low presence in Italian islands (4.8%) and in the southern regions (4.8%) is surprising, given the climatic conditions.

Biogas companies are concentrated in northern Italy, where, after all, national animal husbandry is concentrated. The frequencies observed in the north-western regions are the same as those found in the north-eastern regions, 15 holdings and 38.5% in both areas. On the other side, they are almost entirely absent in southern Italy and only moderately present in central Italy.

The few farms that produce wind energy are concentrated in north-western Italy and in the central regions. Wood-burning production is more widespread in the regions of the North (western with 7 companies, followed by the east with 5 companies) and in those of the Center (5 companies). Energy production from other sources involves 305 farms, located mostly in central Italy (104 holdings, 34.1%), followed by north-eastern Italy (90 holdings, 29.5%), from the regions of southern Italy (51, 16.7%) and from the north-western regions (49, 16.1%).

By altimetric area, 41.2% of the farms producing RE are distributed in the hill, 34.9% in the plain and 23.9% in the mountain. Compared with the distribution of the companies not producing RE, that of the companies producing RE shows a higher percentage of farms in the plain, at the expense of the hill.

Looking at the type of farming of companies producing RE, we find that farms producing crops are mainly located in the hill (49.4%), followed by the plain (34.5%) and the mountain (16.1%). On the contrary, livestock farms producing RE are more concentrated in the plain (37.5%), followed not far by the mountain (35.1%) and by the hill (27.4%). As for the mixed companies that produce RE, the first place is held by the hill (41.3%), followed by the mountain (34.8%) and, finally, by the plain (23.9%).

As far as solar energy is concerned, the farms tend to concentrate in the plain (40.1%) and in the hill (38.7%), while they are poorly present in the mountain (21.3%). Similar results hold for the biogas production, more pronounced in the plain (69.2%), followed by the hill with 23.1%, and last by the mountain with 7.7%. 16 farms produce wind energy; 9 are in the plain (56.3%), 5 in the mountain (31.2%) and 2 in the hill (12.5%), while those involved in wood-burning energy production are 20, 12 in the mountain (60%), 4 in the plain and 4 in the hill. As for the farms that produce energy from other sources, 154 are in the hill (50.5%), 80 in the mountain (26.2%) and 71 in the plain (23.3%).

### EU support

We have briefly analysed the ability of companies to seize the opportunities offered by the CAP (pillars 1 and 2). The support received by a company was aggregated into 4 classes: no aid (0 euros), from 0 to 10,000, from 10,000 to 25,000 and over 25,000.

As we can see from the last two columns of Table 5, reporting the relative distribution of the EU support received by farms not producing and producing RE, farms producing RE generally obtain a higher amount of support, as their distribution is more concentrated in the higher classes. For example, in the highest class (over 25,000 euros) we find 31.1% of farms producing RE, compared to 16.5% of farms not producing RE, while at the other end, only 3% of farms producing RE do not receive any financial support, compared to 8.2% of farms not producing RE.

**Table 5:** Distribution of farms by EU support classes (pillars 1 and 2)

	Pillar 1		Pillar 2		Pillars 1+2	
Classes (euros)	Not prod.	Prod. RE	Not prod.	Prod. RE	Not prod.	Prod. RE
0 (no support)	10.1%	5.2%	51.4%	54.7%	8.2%	2.9%
(0–10,000]	60.3%	53.3%	36.5%	32.3%	52.6%	41.5%
(10,000–25,000]	19.2%	20.2%	8.3%	13.6%	22.7%	24.5%
Over 25,000	10.4%	20.8%	3.7%	9.3%	16.5%	31.1%

A similar remark holds also for the separate distributions of pillars 1 (direct income support) and pillar 2 (support for rural development). In particular, in the case of pillar 2 the number of companies not receiving financial support is higher (44.7% of farms producing RE, 51.4% of farms not producing RE), as expected; moreover, the percentage of companies receiving more than 10,000 euros is 22.9% for farms producing RE, compared to 12.0% of those not producing RE.

In general, the data show that the EU support is widespread and concerns many farms with a relatively small individual amount. Of course, the EU support received may depend on several factors, whose deepening goes being the scope of the present investigation, but the results indeed indicate a greater ability of companies producing RE to take advantage of the CAP opportunities.

### Utilised agricultural area

As regards UAA, 4 types of holdings were identified: small, when the extension does not exceed 5 hectares (ha); medium, when the UAA is comprised between 5 and 15 ha; large, between 15 and 40 ha; and very large, above 40 ha.

**Fig. 1:**
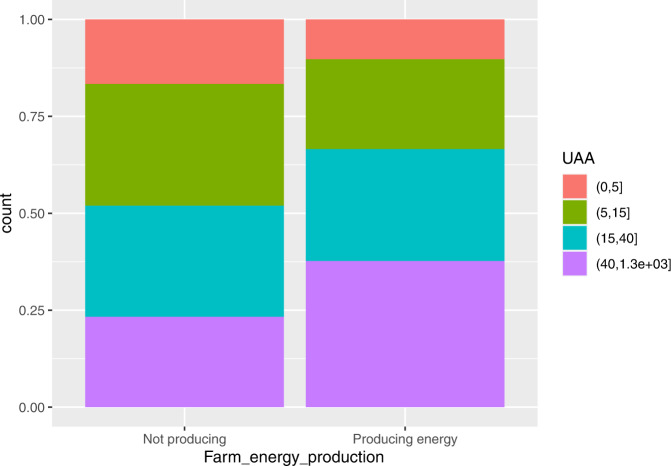
Distribution of farms not producing and producing RE by UAA classes

From Fig. 1 we observe that the holdings in the highest size class (>40 ha) account for 37.7% of farms that produce RE, and only 23.3% of farms that does not produce RE; this result is influenced by the presence of livestock farms of large dimension producing RE (see Fig. 5). Small and medium-sized holdings are less represented, with percentages equal to 10.3% and 23.2%, respectively, in the case of renewable energy production and 16.7% and 31.4%, respectively, in farms not producing RE.

**Fig. 2:**
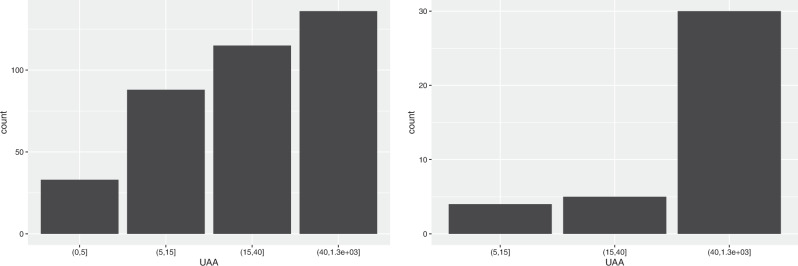
Distribution of farms producing solar energy (on the left) and biogas (on the right) by UAA classes

By energy source, the number of farms producing solar power increases with the size of farms (see Fig. 2); in particular, the solar power production is much less present in small farms.

In the case of biogas production, it is not present in small farms at all, while it is predominant (with 76.9%) in the very large farms (Fig. 2).

Table 6 compares the relative distribution of companies by UAA class for the different types of farming producing/not producing RE. It is confirmed that farms producing RE are generally bigger than the others, in terms of UAA. Moreover, livestock farms are generally bigger than crop farms.

**Table 6:** Distribution of companies by UAA class and type of farming

	Farms not producing RE			Farms producing RE		
UAA classes	Crop	Livestock	Mixed	Crop	Livestock	Mixed
(0.5]	21.5%	5.1%	11.9%	13.7%	4.4%	10.9%
(5.15]	35.6%	21.4%	26.9%	27.8%	16.9%	15.2%
(15.40]	26.6%	33.2%	32.5%	27.1%	29.4%	41.3%
Over 40	18.3%	40.2%	28.7%	31.4%	49.2%	32.6%

### Annual work units

**Fig. 3:**
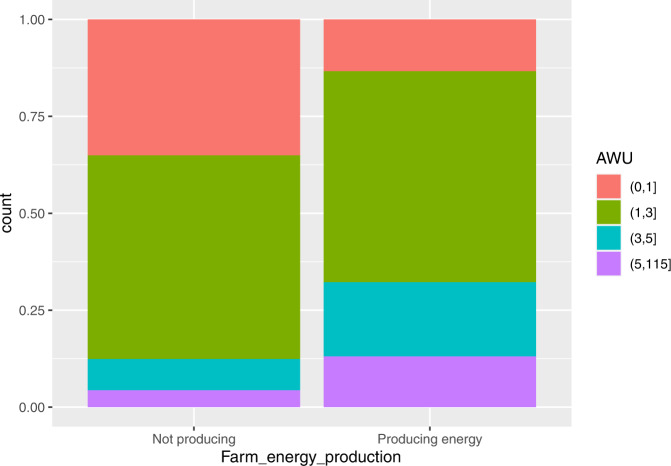
Distribution of farms not producing and producing RE by AWU classes

As for the Annual Work Units, 4 classes were identified: very small (up to 1 AWU), small (1–3 AWU), medium (3–5), large (more than 5 AWU). As we may see from Fig. 3, the small class includes more than 50% of companies, both for farms that do not produce RE (52.5%) and those producing RE (54.4%). On the contrary, the relative distribution for the very small and the larger classes is completely different between the two types of farms: indeed, while the very small farms prevail among the farms not producing RE with 35.1%, the medium and large classes prevail among the farms producing RE with 32.2%. Hence, we may conclude that the renewable energies production employs additional work.

**Fig. 4:**
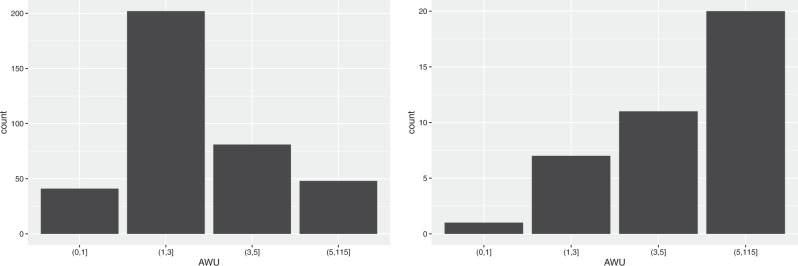
Distribution of farms producing solar energy (on the left) and biogas (on the right) by AWU classes

It is interesting to compare the relative distribution of farms with respect to AWU for the solar and biogas energy production (see Fig. 4): while for biogas the relative number of farms increases markedly with the AWU size, for solar it is the small class (1–3 AWU) the one with the highest relative number.

Table 7 compares the relative distribution of companies by AWU class for the different types of farming producing/not producing RE. More than 50% of companies have a value of AWU comprised between 1 and 3, with slight differences among the different type of farming, both for farms producing and those not producing RE. Moreover, livestock farms are generally bigger than crop farms, in term of working units, for example in the higher UAA class fall 18.3% of crop farms and 40.2% of livestock farms not producing RE, 31.4% and 49.2% of farms producing RE. It is confirmed that farms producing RE generally provide higher full-time employment that the others; besides, livestock farms producing RE require more working time than crop farms.

**Table 7:** Distribution of companies by AWU class and type of farming

	Farms not producing RE			Farms producing RE		
AWU classes	Crop	Livestock	Mixed	Crop	Livestock	Mixed
(0.1]	38.0%	28.4%	31.5%	16.8%	7.7%	13.0%
(1.3]	50.2%	57.0%	60.1%	55.1%	52.0%	60.9%
(3.5]	7.3%	10.3%	5.6%	15.8%	25.4%	15.2%
Over 5	4.5%	4.2%	2.8%	12.2%	14.9%	10.9%

As concerns the share of young (over the age of 40) farmers, we do not observe substantial differences between farmers producing/not producing RE, contrary to our expectation of a higher presence of young. In the case of biogas, we even find that the young farmers are <8%.

### Standard output, total revenues and farm net value added

As regards the Standard Output, 4 size classes were identified: small (8000–50,000), medium (50,000–200,000), large (200,000–500,000) and very large (over 500,000).

As can be seen from Table 8, half of the companies not producing RE are small (48.9%) and 37.0% are medium, while only 14.1% are large or very large. On the contrary, the relative distribution of farms producing RE is more shifted towards the higher SO classes: 32.4% are small, 39.5% are medium, 15.6% are large, and 12.5% are very large. Therefore, the renewable energies production employs additional work, as seen, but in return it provides a higher output.

**Table 8:** Distribution of farms by SO and TR classes

	Standard output (SO)		Total Revenue (TR)	
Classes (euros)	Not prod. RE	Prod. RE	Not prod. RE	Prod. RE
(8000–50,000]	48.9%	32.5%	47.2%	19.7%
(50,000–200,000]	37.0%	39.5%	38.0%	43.6%
(200,000–500,000]	9.8%	15.6%	10.3%	21.1%
Over 500,000	4.3%	12.4%	4.5%	15.6%

**Fig. 5:**
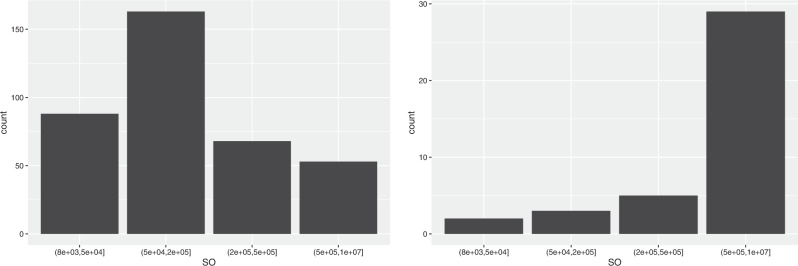
Distribution of farms producing solar energy (on the left) and biogas (on the right) by SO classes

By energy source, from Fig. 5 we note that the farms producing solar power fall for more than 2/3, while half of the farms producing biogas fall in the highest class (48.7%).

The breakdown into classes adopted for the Total Revenues is the same as the one used for the Standard Output: 8000–50,000, 50,000–200,000, 200,000–500,000 and over 500,000. As expected, the distribution of the companies among the classes for TR is similar to that of SO (see Table 8), as are the distributions for farms producing solar and biogas.

The Farm Net Value Added represents the remuneration to fixed factors of production of the farm (work, land, capital) and the remuneration to the entrepreneur’s risks (loss/profit). The FNVA may sometimes be negative (in 1.4% of companies in our sample), and for this reason a “negative value” class had to be added. The “positive value” distribution is displayed in Table 9 for the following classes: 0–20,000 euros (small farms), 20,000–50,000 (medium farms), 50,000–100,000 (large farms), over 100,000 (very large farms).

**Table 9:** Distribution of farms by FNVA classes

Classes (euros)	Not producing RE	Producing RE
Less or equal 0	1.3%	1.8%
(0–20,000]	32.8%	14.2%
(20,000–50,000]	29.4%	22.5%
(50,000–100,000]	18.3%	21.5%
Over 100,000	18.2%	39.9%

The relative distribution of farms producing RE is much more shifted towards the higher values than the distribution of farms not producing RE, and this holds for both crop and livestock farms. Indeed, the very large farms account for 39.9% of farms producing RE, compared to 18.2% of the farms not producing RE. At the other end, only 14.2% of farms producing RE are small, compared to 32.8% of farms not producing RE. On the other hand, especially for biogas production, there still exist technical and economic barriers that make it too costly for small farms to adopt these technologies and prevent them to take advantage of the economies of scale.

**Fig. 6:**
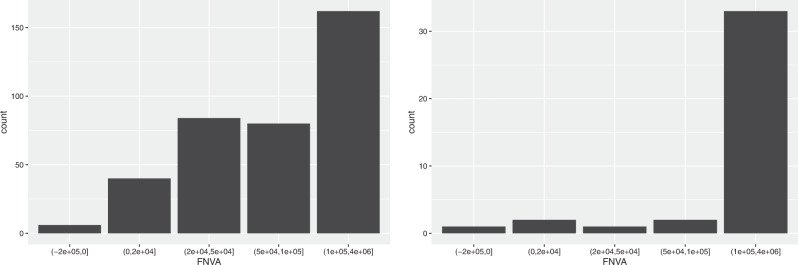
Distribution of farms producing solar energy (on the left) and biogas (on the right) by FNVA classes

By energy source, from Fig. 6 we observe that 43.5% of farms producing solar power fall in the highest class, compared to as many as 84.6% of farms producing biogas. Indeed, the technical dimensions and the adoption costs are less demanding for the solar production, which explains the more balanced farms distribution.

## Economic analysis

### Land and labour productivity

As mentioned in Subsection *Methodology*, we compute the ratios SO/UAA and SO/AWU as proxies of land and labour productivity, respectively.

Table 10 presents the median and mean value of the land and labour productivity ratios by type of farming, compared for farms producing and not producing RE. Both for crop and livestock, farms producing RE exhibit a higher mean standard output per hectare than the other farms. Even more marked is the difference in the mean value of the labour productivity, indicating that in farms producing RE one AWU can produce a higher standard output; this effect is especially relevant for livestock farms producing RE. On the other hand, the differences in the median values are less significant. Of course, the value of the land and labour productivity depends also on several other features that are not always covered by the FADN data and go beyond the aim of the present research.

**Table 10:** Median and mean values of land and labour productivity by type of farming (in euros)

Farms	Median			Mean		
	Crop	Livestock	Mixed	Crop	Livestock	Mixed
*Land productivity*						
All farms	3792	2448	2121	12213	11018	8898
Not producing RE	3794	2338	2115	12175	10805	9126
Producing RE	3723	4079	2244	12800	13172	6953
*Labour productivity*						
All farms	38086	47468	34592	51204	77075	46971
Not producing RE	37663	47216	34438	50366	72632	46925
Producing RE	47923	50914	38258	63892	122002	47366

**Fig. 7:**
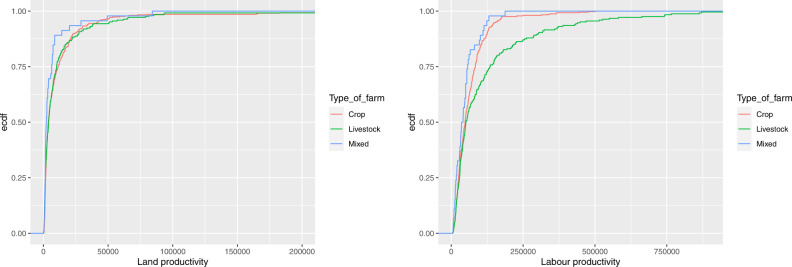
Empirical cumulative distribution functions of land productivity (on the left) and labour productivity (on the right) for farms producing RE by type of farming

It is interesting to compare the empirical cumulative distribution functions (ecdf) of land and labour productivity for farms producing RE (see Fig. 7): while for the land productivity the ecdfs of crop and livestock farms are very close, the difference between the two ecdfs seems much larger for the labour productivity, in accordance with the results of Sharma et al. (1990). To test the statistical significance of the differences among the ecdfs, we have applied the Kolmogorov-Smirnov test of equality of two distributions to all pairs of empirical distributions. According the test results, the only difference that is statistically significant (with a *p* value lower than 0.001) is that between the crop and livestock labour productivity.

In addition, we have seen in the previous section that the size of farms producing RE is generally bigger than that of the other farms, both in terms of UAA and in terms of AWU, with a special emphasis for livestock farms, and this may lead to economies of scale and to a strong bargaining power in the land and labour markets.

Table 11 reports some statistics for the land and labour productivity and allows to compare the mean and median values of farms producing biogas and solar energy to those of the other farms. The results confirm that farms producing biogas provide a much better outcome with respect to both land and labour. As for farms producing solar energy, they, too, obtain a good result, better than the result obtained by farms not producing RE, especially with respect to labour.

**Table 11:** Statistics of the land and labour productivity (in euros)

Farms	Min.	1st.Qu.	Median	Mean	3rd.Qu.	Max.
*Land Productivity*						
All farms	64	1489	3296	11735	8187	6697958
Not producing RE	64	1479	3254	11672	8049	6697958
Producing RE	138	1621	3723	12552	10252	617225
Producing biogas	682	6644	10827	30676	22971	484636
Producing solar energy	300	1979	4565	11940	11316	358376
*Labour Productivity*						
All farms	539	24300	40103	58199	68862	1790763
Not producing RE	539	24227	39584	56279	67455	1156719
Producing RE	4351	26161	48412	83092	89906	1790763
Producing biogas	17265	168517	286159	314027	441191	862528
Producing solar energy	4991	30494	53222	85752	101795	1790763

**Fig. 8:**
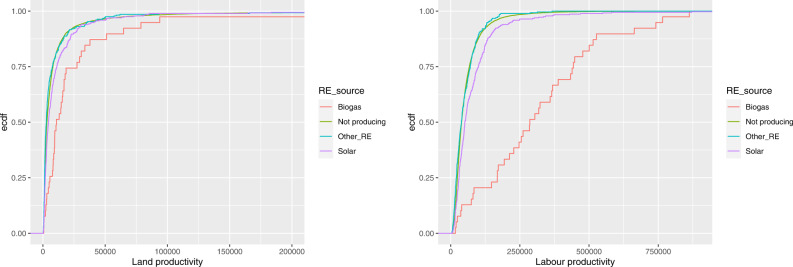
Empirical cumulative distribution functions of land productivity (on the left) and labour productivity (on the right) by source of RE

Figure 8 depicts the ecdfs of land and labour productivity, respectively, for farms producing RE by different sources (biogas, solar, other sources) and compare them to that of farms not producing RE. We observe that the distribution of farms producing RE from other sources (different from solar and biogas) is very close to that of farms not producing RE, while the distributions of farms producing solar energy and, even more, farms producing biogas is shifted to the right, towards higher results. As for the land productivity, the results of the Kolmogorov-Smirnov test of equality of two distributions indicate that the differences are highly statistically significant (with a *p* value lower than 0.001) for all pairs of empirical distributions with the exception of the comparison with the other RE, for which we can accept the equality hypothesis (the *p* value is equal to 0.112). As for the labour productivity, the differences are highly statistically significant for all pairs, with the exception of the comparison with the other RE, for which the difference is less marked (the *p* value is equal to 0.0285).

**Fig. 9:**
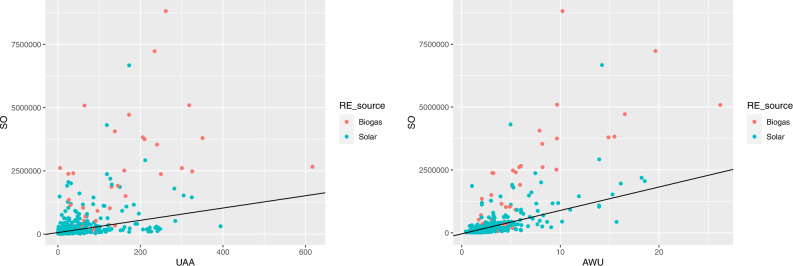
Standard output by utilised agricultural area (on the left) and by annual work units (on the right), for farms producing biogas and solar energy

Figure 9 shows on the left the standard output by utilised agricultural area for farms producing biogas (red points) and solar energy (blue points); the black line represents the average linear relationship between SO and UAA, computed over all the farms. As we may see, most of the farms producing biogas are above the line, with a few having a very high SO; moreover, we observe a wide dispersion of the farms around the line.

Figure 9 displays on the right the standard output by annual work units for farms producing biogas and solar energy, with the black line representing the average linear relationship between SO and AWU, computed over all the farms. Here, the dispersion around the average is smaller.

### Remuneration to the fixed productive factors

As mentioned, we compute the farm net value added per hectare, FNVA/UAA, and the farm net value added per full-time work unit, FNVA/AWU, to verify if the production of energy from renewable sources in agriculture provides a higher (or lower) remuneration to the fixed factors.

Table 12 reports some statistics for the ratios FNVA/UAA and FNVA/AWU and allows us to compare the result obtained by farms producing/not producing RE. By construction, since FNVA may take also negative values (in our sample, in 1.3% of farms not producing RE and 1.8% of farms producing RE), the minimum is negative. The results confirm that farms producing RE, especially those producing biogas, do produce a higher remuneration to the fixed factors, in terms of both FNVA per hectare and FNVA per annual work unit.

**Table 12:** Statistics of the ratios FNVA/UAA and FNVA/AWU (in euros)

Farms	Min.	1st.Qu.	Median	Mean	3rd.Qu.	Max.
*FNVA/UAA*						
All farms	−142356	825	1951	6374	5155	970336
Not producing RE	−142356	816	1902	6278	5016	970336
Producing RE	−8022	989	2512	7621	7684	308393
Producing biogas	−396	2049	3737	9067	8258	83693
Producing solar energy	−8022	1112	2923	7499	7856	250300
*FNVA/AWU*						
All farms	−62807	14177	24766	33789	43374	484871
Not producing RE	−62807	14046	24137	32904	42060	484871
Producing RE	−59147	18414	34042	45261	59139	403427
Producing biogas	−11126	46332	85864	117972	141155	403427
Producing solar energy	−59147	20713	36696	45362	62182	195589

**Fig. 10:**
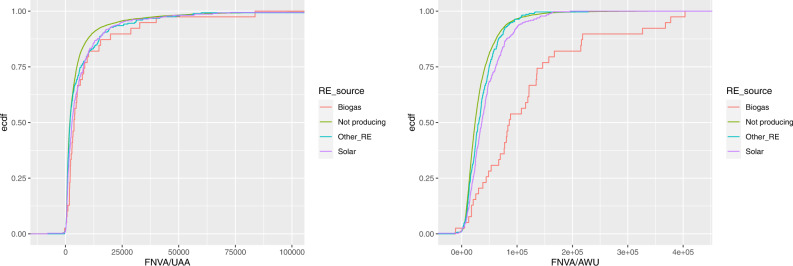
Empirical cumulative distribution functions of FNVA/UAA (on the left) and FNVA/AWU (on the right) by source of RE

Figure 10 displays the ecdfs of FNVA/UAA and FNVA/AWU, respectively, by source of RE and show how much the biogas production is able to raise the value of both ratios; again, the difference with the other farms is especially relevant with respect to the labour. The advantage holds also for the solar power production, even if it is less pronounced. As for the land profitability, the results of the Kolmogorov-Smirnov test indicate that the differences with the distribution of farms not producing RE are highly statistically significant (with a *p* value lower than 0.001) for farms producing biogas and solar, while it is not significant for farms producing other RE (the *p* value is equal to 0.0572). As for the labour profitability, the differences are all highly statistically significant (with a *p* value lower than 0.001).

**Fig. 11:**
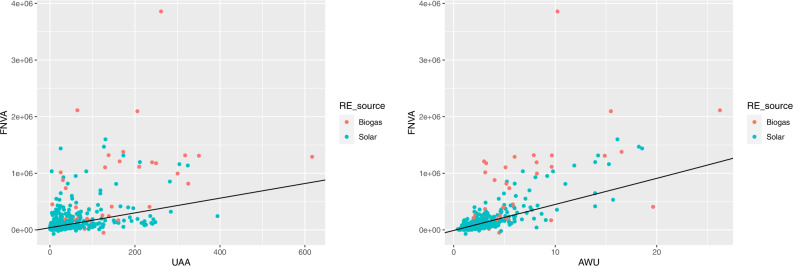
Farm net value added by utilised agricultural area (on the left) and by annual work units (on the right), for farms producing biogas and solar energy

Figure 11 shows the farm net value added by utilised agricultural area and by annual work units, respectively, for farms producing biogas and solar energy. The behaviour of these farms with respect to FNVA is similar to that observed for the SO, as may be expected since both indicators measure the outcome of farms.

## Multivariate regression analysis

In Subsection *Methodology* we have presented the multivariate regression models (1)–(2), that introduce a relationship between the output indicators, SO or FNVA, and the main productive factors simultaneously. The models include also some dummies related to the main features that may impact on the farm output, listed in Table 2.

The results of the model estimation are reported in Tables 13–14. Both models include the productive factors land and work; as was expected, they are highly significant, with a positive effect.

**Table 13:** Results of the regression model on SO

Variable	Coefficient	Estimate	*t* value	Significance
	Intercept	13551.30	1.237	
UAA	UAA	772.36	13.174	***
AWU	AWU	78826.58	76.032	***
RE production: biogas	*d*_1_	1442700.43	35.427	***
RE production: solar	*d*_2_	13346.64	0.993	
RE production: wind	*d*_3_	54450.40	0.866	
RE production: wood	*d*_4_	−88462.16	−1.580	
RE production: other RE	*d*_5_	−28561.76	−1.948	.
Altimetric area: hill	*d*_6_	−33420.42	−5.096	***
Altimetric area: mountain	*d*_7_	−83838.04	−11.513	***
Type of farming: livestock	*d*_8_	84380.77	13.422	***
Type of farming: mixed	*d*_9_	7739.42	0.624	
Management system	*d*_10_	108306.41	7.832	***
Educational level: upper secondary	*d*_11_	17610.01	3.185	**
Educational level: tertiary	*d*_12_	54248.31	6.140	***
Age	*d*_13_	−11097.26	−1.455	
EU support: (0,10] thousand €	*d*_14_	−18779.11	−1.914	.
EU support: (10,25] thousand €	*d*_15_	−28069.71	−2.576	*
EU support: > 25 thousand €	*d*_16_	17367.87	1.393	
Geographical area: Centre	*d*_17_	−71250.21	−8.189	***
Geographical area: Islands	*d*_18_	−91454.95	−9.216	***
Geographical area: North-East	*d*_19_	−16112.55	−2.018	*
Geographical area: South	*d*_20_	−77971.89	−10.522	***
Significance of regression				
Adjusted R-squared	0.5511			
F-statistic	554.8			
*p* value	<2.2e−16			

Significance codes: *** 0.001, ** 0.01, * 0.05, . 0.1

**Table 14:** Results of the regression model on FNVA

Variable	Coefficient	Estimate	*t* value	Significance
	Intercept	9336.93	1.867	.
UAA	UAA	518.75	19.380	***
AWU	AWU	40339.74	85.221	***
RE production: biogas	*d*_1_	372798.14	20.050	***
RE production: solar	*d*_2_	11487.76	1.872	.
RE production: wind	*d*_3_	−38412.59	−1.338	
RE production: wood	*d*_4_	−33578.03	−1.313	
RE production: other RE	*d*_5_	1986.04	0.297	
Altimetric area: hill	*d*_6_	−8842.04	−2.953	**
Altimetric area: mountain	*d*_7_	−23638.07	−7.109	***
Type of farming: livestock	*d*_8_	18628.25	6.490	***
Type of farming: mixed	*d*_9_	−6431.30	−1.136	
Management system	*d*_10_	43842.30	6.943	***
Educational level: upper secondary	*d*_11_	8351.18	3.308	***
Educational level: tertiary	*d*_12_	20011.76	4.961	***
Age	*d*_13_	−2722.07	−0.782	
EU support: (0,10] thousand €	*d*_14_	−6466.56	−1.443	
EU support: (10,25] thousand €	*d*_15_	−11536.35	−2.318	*
EU support: > 25 thousand €	*d*_16_	17818.77	3.130	**
Geographical area: Centre	*d*_17_	−39879.37	−10.038	***
Geographical area: Islands	*d*_18_	−44151.12	−9.745	***
Geographical area: North-East	*d*_19_	−19952.37	−5.474	***
Geographical area: South	*d*_20_	−40170.96	−11.873	***
Significance of regression				
Adjusted R-squared	0.5783			
F-statistic	619.6			
*p* value	<2.2e−16			

Significance codes: *** 0.001, ** 0.01, * 0.05, . 0.1

With regard to the impact of the production of renewable energy, we may see that this impact is positive and highly significant only for biogas, both for SO and FNVA, while it is significant at a 10% level for solar only in the FNVA model. These results are in accordance with the analysis carried out in the previous sections, which have already pointed out the advantage of farms producing biogas, and take into account the effect of size, captured by the coefficients of UAA and AWU.

As for the impact of the altimetric area on the output indicators, the results show that farms located on the hill and on the mountains are at a disadvantage, compared to farms located on the plain: the estimated coefficients are always negative and highly significant in both models, with a higher output decrease in the mountain.

As regards the type of farming, we may see that livestock farms present on average a higher output than crop farms, with regard to both SO and FNVA: the estimated coefficient is highly significant and positive. On the contrary, the estimated coefficient associated to mixed farms is never significant.

The management system is highly significant in both models with a positive coefficient, implying that the direct management system pays more than the others. As for the educational level, it is highly significant with a positive effect, which is higher for the tertiary level, as can be expected. On the contrary, the age (over/under 40) is not significant in either model.

On the other hand, the effect of the EU support on the economic output is somewhat controversial and, on the whole, not clear-cut: in the SO model, only the parameter of the (10,25] class is significant and negative, in the FNVA model both the (10,25] and the >25 classes are significant, with opposite signs.

The geographical area does affect the economic output, since the parameters are all highly significant, and suggests that the North-West area exhibits the highest result, followed by the North-East.

We have also estimated the reduced regression models without the non significant variables, obtaining similar results.

## Conclusions

For the production of renewable energy considerable financial resources have been allocated, and many more will be in next years. Despite the huge resources available, however, there are many barriers that hinder the production and consequent use of REs in agriculture. According to the final report of the EIP-AGRI (2019), the financial, technical, social and regulatory issues related to natural resources represent the main bottlenecks. Furthermore, RE technologies are still expensive and require new skills, regulations do not always support the sale of the excess electricity produced and the procedures are complex (Abanades et al. (2022)). What’s more, Italy’s dependence on import for both raw materials and plant components associate additional risks of temporary scarsity and price volatility (Cai et al. (2017)), which may cause sudden rises in the plant costs. For the growth of renewable energies on farms, in addition to financial support, efforts to train and develop capacity need to be further incentivized. Furthermore, information on successful experiences on the use and production of renewable energy should be shared in order to show farmers the concrete benefits of renewable energy in terms of economic results.

With regard to this, our investigation shows that few farms in the FADN sample produce energy from renewable sources in Italy. The economic results, however, have shown, especially in the case of biogas and solar energy production, how this additional activity provides superior economic results in companies that produce RE, compared to those that do not. Moreover, the educational level is on average higher, as can be expected, while the share of young farmers is not substantially different, contrary to what could be expected (Salvioni et al. (2020), Tate et al. (2012)).

In the case of solar energy, in Italy there is a legislation that limits conflicts in land use and which, indirectly, favours food security over energy security. However, the regions where such production is scarcely practiced by farms are those where this form of energy is massively produced by the industrial sector. Plants that produce solar energy in agricultural areas are not prohibited but they are not incentivized either. As a matter of fact, not to set limits to the use of agricultural land brings risks to the natural heritage protection and to food security; from this point of view, the use of abandoned and neglected areas could be an excellent solution (Martinho (2018)).

The case of biogas is specially interesting, given the size and spread of livestock farms in Italy, especially in northern Italy. The structure of farms in Italy is characterised by small and medium-sized enterprises, that may not find it convenient to invest in a plant for the treatment of livestock waste, which on the other hand is essential for compliance with the provisions of the nitrate’s directive. In 2020, the European Commission sent Italy a second letter of formal notice, after the one sent in November 2018, for failure to comply with this directive, aimed at preventing water and soil pollution caused by nitrates of agricultural origin. It is thus of the foremost importance to be able to push a higher number of farms to implement biogas production (Hansson (2008), Dimoudi et al. (2017)). This calls for a strong encouragement of farmers to participate in collective biogas investment, thus overcoming the obstacle posed by the small size of most farms; and the results of our analysis show that it may also be economically convenient.

Some additional suggestions for policy makers can be listed, in order to increase the RE production in the agricultural sector:

- Support and finance information, training, technical assistance and consultancy actions to encourage the production of renewable energy, including successful examples in terms of profitability and/or profitability.
- Encourage the adoption and diffusion of advanced management systems, through the use of innovative technologies that promote the efficiency of RE production in agriculture.
- Combine funding from different sources and provide a guide to farmers on the different overly fragmented opportunities, dispersed in different measures; from the policy makers point of view, a reorganisation of all these measures could be appropriate.
- Promote the circular economy in the reuse of raw materials and by-products, mainly deriving from processes in the agro-livestock sector, including their logistical and organisational innovation. After a decade of steadily declining costs, the renewable energy sector is experiencing a turning point, due to shipping delays and rising raw material costs, mostly involving solar. These supply constraints could slow the development of solar power production in the world and in Europe in particular.

In addition, with special reference to Italy:

- Provide specific support for small farms and encourage the association of agricultural businesses in the construction of plants, thus allowing to make investments not within the reach of small farms.
- Reorganise and simplify the rigid and extremely complex institutional framework and the fragmented support system.

As for further research suggestions, this study could be generalised in several directions, to explore some issues that are not tackled in the present contribution and require additional information: to analyse other European countries with FADN data sets, to compare the economic outcome of other sources of RE, to investigate the economic results produced using different technologies for the production of RE, to adopt a cost-benefit analysis approach, with the aim of identifying the underlying business model, to further investigate the effectiveness of the various (EU, national or regional) support grants on the different RE produced in agriculture, with special attention to the CAP instruments.

A different methodology that is worth exploring in future research is a two stage model, in which the willingness to adopt the RE production can be modeled in the first stage. In order to adopt such an approach for Italian farms, it will be necessary to carry out a preliminary survey investigating this willingness in Italy; see, for example, the paper Burg et al. (2021) that uses a survey for biogas facilities in Switzerland.

Another interesting research development is the construction of an efficient frontier, which can be carried out using a non parametric methodology such as Data Envelopment Analysis (DEA). A DEA approach would allow to identify the most efficient farms, representing the best practices, and to compute a relative efficiency score for the inefficient ones.

## Data Availability

The datasets analysed during the current study are available in the RICA (the Italian FADN) repository, https://rica.crea.gov.it/.

## Footnotes

1 The authors wish to thank CREA (Consiglio per la Ricerca in Agricoltura e l’Analisi dell’Economia Agraria) for providing access to RICA data base, the Italian FADN.

## References

[CR1] Abanades S, Abbaspour H, Ahmadi A, Das B, Ehyaei MA (2022). A critical review of biogas production and usage with legislations framework across the globe.. Int J Environ Sci Technol.

[CR2] Ball VE, Färe R, Grosskopf S, Margaritis D (2015). The role of energy productivity in U.S. agriculture. Energy Econ.

[CR3] Ballarin A, Vecchiato D, Tempesta T, Marangon F, Troiano S (2011). Biomass energy production in agriculture: A weighted goal programming analysis. Energy Policy.

[CR4] Bartolini F, Gava O, Brunori G (2017). Biogas and EU’s 2020 targets: Evidence from a regional case study in Italy. Energy Policy.

[CR5] Blazejczy-Majka L, Kal R, Maciejewski K (2012). Productivity and efficiency of large and small field crop farms and mixed farms of the old and new EU regions. Agric Econ.

[CR6] Burg V, Troitzsch KG, Akyol D, Baier U, Hellweg S, Thees O (2021). Farmer’s willingness to adopt private and collective biogas facilities: An agent-based modeling approach. Resour Conserv Recycling.

[CR7] Cai M, Cusumano N, Lorenzoni A, Pontoni F (2017). A comprehensive ex-post assessment of RES deployment in Italy: Jobs, value added and import leakages. Energy Policy.

[CR8] Carrosio G (2014). Production from biogas in the Italian countryside: Modernization vs. repeasantization. Biomass Bioenergy.

[CR9] Chel A, Kaushik G (2011). Renewable energy for sustainable agriculture. Agron Sustain Dev.

[CR10] Coppola A, Scardera A, Tosco D (2013) Economic profitability and long-term viability in Italian Agriculture. Politica Agric. Internazionale 71–84

[CR11] Coppola A, Scardera A, Amato M, Verneau F (2020). Income Levels and Farm Economic Viability in Italian Farms: An Analysis of FADN Data. Sustainability.

[CR12] Di Nucci MR, Prontera A, (2021). The Italian energy transition in a multilevel system: between reinforcing dynamics and institutional constraints. Z Politikwiss. 10.1007/s41358-021-00306-y

[CR13] Dimoudi A, Stathis V, Pallas C, (2017). Transformation of a Small-Livestock, Rural Community into a Green, Nearly-Zero CO2-Emissions Settlement. In: Bisello A, Vettorato D, Stephens R, Elisei P (eds) Smart and Sustainable Planning for Cities and Regions. SSPCR 2015. Green Energy and Technology. Springer, Cham. 10.1007/978-3-319-44899-2_19

[CR14] Dorward A (2013). Agricultural labour productivity, food prices and sustainable development impacts and indicators. Food Policy.

[CR15] EIP (European Innovation Partnership) -AGRI, (2019). Focus Group Final report Focus group Renewable energy on the farm. Final report. https://ec.europa.eu/eip/agriculture/sites/default/files/eip-agri_fg_renewable_energy_on_the_farm_final_report_2019_en.pdf.

[CR16] ENEA (Agenzia Nazionale Efficienza Energetica) (2018) Rapporto annuale efficienza energetica. Analisi e risultatidelle policy di efficienza energetica nel nostro paese. https://www.enea.it/it/seguici/pubblicazioni/pdfvolumi/2018/raee_2018.pdf.

[CR17] European Commission, (2018). In-depth analysis in support of the commission, communication com(2018) 773, A Clean Planet for all, A European long-term strategic vision for a prosperous, modern, competitive and climate neutral economy. https://ec.europa.eu/clima/sites/default/files/docs/pages/com_2018_733_analysis_in_support_en_0.pdf).

[CR18] European Commission, (2019). Communication from the Commission to the European Parliament, the European Council, the Council, the European Economic and Social Committee and the Committee of the Regions The European Green Deal available at: https://eur-lex.europa.eu/resource.html?uri=cellar:b828d165-1c22-11ea-8c1f01aa75ed71a1.0002.02/DOC_1&format=PDF).

[CR19] European Commission, (2020). Communication from the commission to the European Parliament, the Council, the European economic and social committee and the Committee of the Regions, A Farm to Fork Strategy for a fair,healthy and environmentally-friendly food system, COM/2020/381 final. https://eur-lex.europa.eu/resource.html?uri=cellar:ea0f9f73-9ab2-11ea-9d2d-01aa75ed71a1.0001.02/DOC_1&format=PDF.

[CR20] European Parliament, (2020). Renewable energy. https://www.europarl.europa.eu/factsheets/en/sheet/70/renewable-energy).

[CR21] EPRS, (2016). Renewable energy in EU agriculture, EPRS (European Parliamentary Research Service), https://ec.europa.eu/eurostat/web/energy/data/shares.

[CR22] Eyuboglu K, Uzar U (2020). Examining the roles of renewable energy consumption and agriculture on CO2 emission in lucky-seven countries. Environ Sci Pollut Res.

[CR23] Fernandez-Perez A, Frijns B, Tourani-Rad A (2016). Contemporaneous interactions among fuel, biofuel and agricultural commodities. Energy Econ.

[CR24] Ge J, Sutherland L-A, Polhill JG, Matthews K, Miller D, Wardell-Johnson D (2017). Exploring factors affecting on-farm renewable energy adoption in Scotland using large-scale microdata. Energy Policy.

[CR25] Giannakis E, Bruggeman A (2018) Exploring the labour productivity of agricultural systems across European regions: A multilevel approach. Land Use Policy 77:94-106

[CR26] GSE (2019) Energia da fonti rinnovabili in Italia - Rapporto Statistico. GSE Editions. https://www.gse.it/documenti_site/Documenti%20GSE/Rapporti%20statistici/GSE%20-%20Rapporto%20Statistico%20FER%202018.pdf.

[CR27] GSE (2020) Fonti rinnovabili in Italia e in Europa 2018. https://www.gse.it/documenti_site/Documenti%20GSE/Rapporti%20statistici/GSE%20-%20Fonti%20rinnovabili%20in%20Italia%20e%20in%20Europa%20-%202018.pdf.

[CR28] Gorton M, Davidova S, Ratinger T, Zawalinska K, Iraizoz B (2005). Farm Productivity and Profitability: A Comparative Analysis of Selected New and Existing EU Member States1. Comparative Econ Studies.

[CR29] Hansson H (2008). Are larger farms more efficient? A farm level study of the relationships between efficiency and size on specialized dairy farms in Sweden. Agric Food Sci.

[CR30] IEA, (2019) World Energy Outlook 2019. IEA, Paris https://www.iea.org/reports/world-energy-outlook-2019.

[CR31] IEA, (2020a) Renewable Information: overview. https://www.iea.org/reports/renewables-information-overview.

[CR32] IEA, (2020b) Outlook for biogas and biomethane: Prospects for organic growth. https://www.iea.org/reports/outlook-for-biogas-and-biomethane-prospects-for-organic-growth/sustainable-supply-potential-and-costs.

[CR33] IEA, (2021) World Energy Outlook 2021. https://www.iea.org/weo.

[CR34] IRENA and FAO, (2021) Renewable energy for agri-food systems – Towards the Sustainable Development Goals and the Paris agreement. Abu Dhabi and Rome. 10.4060/cb7433en

[CR35] ISPRA, (2020) Factors of greenhouse gas emissions into the atmosphere in the national electricity sector and in the main European countries. https://www.isprambiente.gov.it/files2020/pubblicazioni/rapporti/Rapporto317_2020.pdf.

[CR36] Leonardo WJ, van de Ven GWJ, Udo H (2015). Labour not land constrains agricultural production and food self-sufficiency in maize-based smallholder farming systems in Mozambique. Food Sec.

[CR37] Liu X, Zhang S, Bae J (2017). The impact of renewable energy and agriculture on carbon dioxide emissions: investigating the environmental Kuznets curve in four selected ASEAN countries. J Clean Prod.

[CR38] Marongiu S, Cesaro L (2017) Economic performance and profitability of agricultural holdings in Inner Areas. Italian J Planning Pract 7:100–124

[CR39] Martinho VJPD (2018). Interrelationships between renewable energy and agricultural economics: An overview. Energy Strategy Rev.

[CR40] Ministero dello Sviluppo Economico (2010) Decreto 10 settembre 2010, Linee guida per l’autorizzazione degli impianti alimentati da fonti rinnovabili. GU Serie Generale n.219 del 18-09-2010. https://www.gazzettaufficiale.it/eli/id/2010/09/18/10A11230/sg.

[CR41] MISE, MATTM, MIT, (2019) Piano nazionale integrato per l’energia e il clima. Ministero dello SviluppoEconomico. Ministero dell’Ambiente e della Tutela del Territorio e del Mare, Ministero delle Infrastrutture e dei Trasporti. https://www.mise.gov.it/images/stories/documenti/PNIEC_finale_17012020.pdf UN (2018) The 17 goals - Sustainable development goals. https://sdgs.un.org/goals.

[CR42] Musacchio A, Re V, Mas-Pla J (2020). EU Nitrates Directive, from theory to practice: Environmental effectiveness and influence of regional governance on its performance. Ambio.

[CR43] Pirelli T, Chiumenti A, Morese MM, Bonati G, Fabiani S, Pulighe G (2021). Environmental sustainability of the biogas pathway in Italy through the methodology of the Global Bioenergy Partnership. J Cleaner Prod.

[CR44] Popp J, Lakner Z, Harangi-Rákos M, Fári M (2014). The effect of bioenergy expansion: Food, energy, and environment. Renewable Sustai Energy Rev.

[CR45] Rikkonen P, Tapio P, Rintamäki H (2019). Visions for small-scale renewable energy production on Finnish farms - A Delphi study on the opportunities for new business. Energy Policy.

[CR46] Rocca UV, (2021) Impianti fotovoltaici su terreni agricoli. Rinnovabili.it - Il quotidiano sulla sostenibilità ambientale. https://www.rinnovabili.it/energia/fotovoltaico/impianti-fotovoltaici-terreni-agricoli/

[CR47] Rokicki T, Perkowska A, Klepacki B, Bórawski P, Bełdycka-Bórawska A, Michalski K (2021). Changes in Energy Consumption in Agriculture in the EU Countries. Energies.

[CR48] Salvioni C, Henke R, Vanni F (2020). The impact of non-agricultural diversification on financial performance: Evidence from family farms in Italy. Sustainability.

[CR49] Sharma KC, Rao DP, Shepherd W (1990). Productivity of Agricultural Labour and Land: An International Comparison. Agric Econ.

[CR50] Tate G, Mbzibain A, Ali S (2012). A comparison of the drivers influencing farmers’ adoption of enterprises associated with renewable energy. Energy Policy.

[CR51] Taylor K, Iorio V (2021) Rinnovabili, la bozza della nuova direttiva Ue delude il settore geotermico. EURACTIV Italia. https://euractiv.it/section/energia/news/rinnovabili-la-bozza-della-nuova-direttiva-ue-delude-il-settore-geotermico/.

[CR52] UN (2018) The 17 goals – Sustainable development goals.

[CR53] Waheed R, Chang D, Sarwar S, Chen W (2018). Forest, agriculture, renewable energy, and CO2 emission. J Cleaner Produc.

[CR54] Wang Y, Wu C, Yang L (2014). Oil price shocks and agricultural commodity prices. Energy Econ.

[CR55] Zygierewicz A and Salvador Sanz L (2021) Renewable Energy Directive Revision of Directive (EU) 2018/2001. EPRS (European Parliamentary Research Service) Ex-Post Evaluation Unit PE 662.619. https://www.europarl.europa.eu/RegData/etudes/BRIE/2021/662619/EPRS_BRI(2021)662619_EN.pdf).

